# Supplementary material for: Limited potexvirus diversity in eastern Gulf of Mexico seagrass meadows
Source: J Gen Virol. 2024 Jun 18;105(6):002004. doi: 10.1099/jgv.0.002004 (PMC11256451; doi:10.1099/jgv.0.002004)
Supplement: Uncited Table S1. [file jgv-105-02004-s001.pdf]

Table S1. Metadata of seagrass samples used in the RT-PCR survey. Samples successfully amplified by Potex5F/Potex2-RC primers are highlighted in bold and the NCBI accession numbers of their amplicon sequences are listed in the last column.

Sample ID	State, Country	Region	Collection Date	Site Description	Latitude	Longitude	Species	Accession
**S1T5 Tt**	**FL, USA**	**Upper Tampa Bay**	**4-Feb-22**	**S1T5 - Old Tampa Bay**	**27.95788**	**-82.54663**	***Thalassia testudinum***	**OR827692**
S1T5 Sf	FL, USA	Upper Tampa Bay	4-Feb-22	S1T5 - Old Tampa Bay	27.95788	-82.54663	*Syringodium filiforme*	
S1T5 Rm	FL, USA	Upper Tampa Bay	4-Feb-22	S1T5 - Old Tampa Bay	27.95788	-82.54663	*Ruppia maritima*	
S1T16 Tt	FL, USA	Upper Tampa Bay	4-Feb-22	S1T16 - Old Tampa Bay	27.88228	-82.62015	*Thalassia testudinum*	
S1T16 Sf	FL, USA	Upper Tampa Bay	4-Feb-22	S1T16 - Old Tampa Bay	27.88228	-82.62015	*Syringodium filiforme*	
S1T16 Rm	FL, USA	Upper Tampa Bay	4-Feb-22	S1T16 - Old Tampa Bay	27.88228	-82.62015	*Ruppia maritima*	
S1T16 Hw	FL, USA	Upper Tampa Bay	4-Feb-22	S2T2 - Hillsborough Bay	27.82347	-82.39305	*Halodule wrightii*	
S1T16 Rm	FL, USA	Upper Tampa Bay	4-Feb-22	S2T2 - Hillsborough Bay	27.82347	-82.39305	*Ruppia maritima*	
S3T8 Tt 2021	FL, USA	Upper Tampa Bay	26-Jan-22	S3T8 - Lassing Park	27.75327	-82.63035	*Thalassia testudinum*	
S3T8 Sf 2021	FL, USA	Upper Tampa Bay	26-Jan-22	S3T8 - Lassing Park	27.75327	-82.63035	*Syringodium filiforme*	
S3T8 Rm 2021	FL, USA	Upper Tampa Bay	26-Jan-22	S3T8 - Lassing Park	27.75327	-82.63035	*Ruppia maritima*	
**S3T8 Tt 2023**	**FL, USA**	**Upper Tampa Bay**	**3-Oct-23**	**S3T8 - Lassing Park**	**27.75327**	**-82.63035**	***Thalassia testudinum***	**OR879052**
S3T8 Sf 2023	FL, USA	Upper Tampa Bay	26-Jan-22	S3T8 - Lassing Park	27.75327	-82.63035	*Syringodium filiforme*	
S3T8 Rm 2023	FL, USA	Upper Tampa Bay	26-Jan-22	S3T8 - Lassing Park	27.75327	-82.63035	*Ruppia maritima*	
S3T13 Tt	FL, USA	Upper Tampa Bay	4-Feb-22	S3T13 - Apollo Beach	27.7566	-82.44758	*Thalassia testudinum*	
S3T13 Hw	FL, USA	Upper Tampa Bay	4-Feb-22	S3T13 - Apollo Beach	27.7566	-82.44758	*Halodule wrightii*	
S4T5 Tt	FL, USA	Lower Tampa Bay	26-Jan-22	S4T5 - Terra Ceia Bay	27.54417	-82.60615	*Thalassia testudinum*	
S4T5 Sf	FL, USA	Lower Tampa Bay	26-Jan-22	S4T5 - Terra Ceia Bay	27.54417	-82.60615	*Syringodium filiforme*	
S4T5 Hw	FL, USA	Lower Tampa Bay	26-Jan-22	S4T5 - Terra Ceia Bay	27.54417	-82.60615	*Halodule wrightii*	
S4T5 Rm	FL, USA	Lower Tampa Bay	26-Jan-22	S4T5 - Terra Ceia Bay	27.54417	-82.60615	*Ruppia maritima*	
S4T10 Tt	FL, USA	Lower Tampa Bay	26-Jan-22	S4T10 - Lower Tampa Bay	27.52134	-82.65712	*Thalassia testudinum*	
S4T10 Sf	FL, USA	Lower Tampa Bay	26-Jan-22	S4T10 - Lower Tampa Bay	27.52134	-82.65712	*Syringodium filiforme*	
S4T10 Hw	FL, USA	Lower Tampa Bay	26-Jan-22	S4T10 - Lower Tampa Bay	27.52134	-82.65712	*Halodule wrightii*	
S4T10 Rm	FL, USA	Lower Tampa Bay	26-Jan-22	S4T10 - Lower Tampa Bay	27.52134	-82.65712	*Ruppia maritima*	
BCB11 Tt	FL, USA	Lower Tampa Bay	26-Jan-22	BCB11 - Boca Ciega Bay	27.72243	-82.69762	*Thalassia testudinum*	
BCB11 Sw	FL, USA	Lower Tampa Bay	26-Jan-22	BCB11 - Boca Ciega Bay	27.72243	-82.69762	*Syringodium filiforme*	

BCB11 Hw	FL, USA	Lower Tampa Bay	26-Jan-22	BCB11 - Boca Ciega Bay	27.72243	-82.69762	*Halodule wrightii*	
27	FL, USA	Lower Tampa Bay	1-Aug-22	Terra Ceia Aquatic Preserve	27.58404412	-82.61651603	*Thalassia testudinum*	OR827699
28	FL, USA	Lower Tampa Bay	1-Aug-22	Terra Ceia Aquatic Preserve	27.58404505	-82.61643497	*Thalassia testudinum*	PP430550
29	FL, USA	Lower Tampa Bay	1-Aug-22	Terra Ceia Aquatic Preserve	27.58404597	-82.61635392	*Thalassia testudinum*	OR827693
37	FL, USA	Lower Tampa Bay	1-Aug-22	Terra Ceia Aquatic Preserve	27.58397095	-82.61659599	*Thalassia testudinum*	OR827703
48	FL, USA	Lower Tampa Bay	1-Aug-22	Terra Ceia Aquatic Preserve	27.5838997	-82.61651385	*Thalassia testudinum*	OR827694
49	FL, USA	Lower Tampa Bay	1-Aug-22	Terra Ceia Aquatic Preserve	27.58390062	-82.61643288	*Thalassia testudinum*	PP430551
50	FL, USA	Lower Tampa Bay	1-Aug-22	Terra Ceia Aquatic Preserve	27.58390163	-82.61635182	*Thalassia testudinum*	PP430552
51	FL, USA	Lower Tampa Bay	1-Aug-22	Terra Ceia Aquatic Preserve	27.58390255	-82.61627077	*Thalassia testudinum*	PP430553
52	FL, USA	Lower Tampa Bay	1-Aug-22	Terra Ceia Aquatic Preserve	27.58390347	-82.6161898	*Thalassia testudinum*	PP430554
53	FL, USA	Lower Tampa Bay	1-Aug-22	Terra Ceia Aquatic Preserve	27.5839044	-82.61610875	*Thalassia testudinum*	OR827695
54	FL, USA	Lower Tampa Bay	1-Aug-22	Terra Ceia Aquatic Preserve	27.5839054	-82.61602778	*Thalassia testudinum*	PP430555
62	FL, USA	Lower Tampa Bay	1-Aug-22	Terra Ceia Aquatic Preserve	27.5838303	-82.61626977	*Thalassia testudinum*	PP430556
64	FL, USA	Lower Tampa Bay	1-Aug-22	Terra Ceia Aquatic Preserve	27.58383223	-82.61610774	*Thalassia testudinum*	OR827702
66	FL, USA	Lower Tampa Bay	1-Aug-22	Terra Ceia Aquatic Preserve	27.58383407	-82.61594564	*Thalassia testudinum*	OR827697
67	FL, USA	Lower Tampa Bay	1-Aug-22	Terra Ceia Aquatic Preserve	27.58383508	-82.61586467	*Thalassia testudinum*	PP430557
74	FL, USA	Lower Tampa Bay	1-Aug-22	Terra Ceia Aquatic Preserve	27.58375813	-82.61626868	*Thalassia testudinum*	PP430558
75	FL, USA	Lower Tampa Bay	1-Aug-22	Terra Ceia Aquatic Preserve	27.58375905	-82.61618771	*Thalassia testudinum*	PP430559
77	FL, USA	Lower Tampa Bay	1-Aug-22	Terra Ceia Aquatic Preserve	27.58376098	-82.6160256	*Thalassia testudinum*	PP430560
78	FL, USA	Lower Tampa Bay	1-Aug-22	Terra Ceia Aquatic Preserve	27.5837619	-82.61594463	*Thalassia testudinum*	PP430561
79	FL, USA	Lower Tampa Bay	1-Aug-22	Terra Ceia Aquatic Preserve	27.58376283	-82.61586358	*Thalassia testudinum*	PP430562
80	FL, USA	Lower Tampa Bay	1-Aug-22	Terra Ceia Aquatic Preserve	27.58376383	-82.61578253	*Thalassia testudinum*	PP430563
84	FL, USA	Lower Tampa Bay	1-Aug-22	Terra Ceia Aquatic Preserve	27.58368689	-82.61618662	*Thalassia testudinum*	PP430564
88	FL, USA	Lower Tampa Bay	1-Aug-22	Terra Ceia Aquatic Preserve	27.58369066	-82.61586249	*Thalassia testudinum*	PP430565
93	FL, USA	Lower Tampa Bay	1-Aug-22	Terra Ceia Aquatic Preserve	27.58361564	-82.61610456	*Thalassia testudinum*	OR827698
96	FL, USA	Lower Tampa Bay	1-Aug-22	Terra Ceia Aquatic Preserve	27.58361849	-82.61586148	*Thalassia testudinum*	PP430566
97	FL, USA	Lower Tampa Bay	1-Aug-22	Terra Ceia Aquatic Preserve	27.58361941	-82.61578043	*Thalassia testudinum*	OR827705
101	FL, USA	Lower Tampa Bay	1-Aug-22	Terra Ceia Aquatic Preserve	27.58354439	-82.61602242	*Thalassia testudinum*	OR879054
105	FL, USA	Lower Tampa Bay	1-Aug-22	Terra Ceia Aquatic Preserve	27.58354817	-82.61569837	*Thalassia testudinum*	OR827704
119	FL, USA	Lower Tampa Bay	1-Aug-22	Terra Ceia Aquatic Preserve	27.58340475	-82.61561522	*Thalassia testudinum*	PP430548
128	FL, USA	Lower Tampa Bay	1-Aug-22	Terra Ceia Aquatic Preserve	27.58325941	-82.6156941	*Thalassia testudinum*	OR879053

**132**	**FL, USA**	**Lower Tampa Bay**	**1-Aug-22**	**Terra Ceia Aquatic Preserve**	**27.58318816**	**-82.61561204**	***Thalassia testudinum***	**PP430549**
**A1**	**FL, USA**	**Dry Tortugas National Park**	**20-May-22**	**Key West**	**24.581857**	**-81.799379**	***Thalassia testudinum***	**PP430567**
**A2**	**FL, USA**	**Dry Tortugas National Park**	**20-May-22**	**Key West**	**24.581865**	**-81.799295**	***Thalassia testudinum***	**PP430568**
L1	FL, USA	Dry Tortugas National Park	19-May-22	Marquesas Key	24.560963	-82.125589	*Thalassia testudinum*	
**L2**	**FL, USA**	**Dry Tortugas National Park**	**19-May-22**	**Marquesas Key**	**24.560981**	**-82.12555**	***Thalassia testudinum***	**PP430569**
**Q1**	**FL, USA**	**Dry Tortugas National Park**	**17-May-22**	**Garden Key**	**24.626714**	**-82.874075**	***Thalassia testudinum***	**PP430570**
**Q2**	**FL, USA**	**Dry Tortugas National Park**	**17-May-22**	**Garden Key**	**24.626673**	**-82.874162**	***Thalassia testudinum***	**PP430571**
**R**	**FL, USA**	**Dry Tortugas National Park**	**16-May-22**	**Bush Key**	**24.626357**	**-82.868817**	***Thalassia testudinum***	**OR879056**
Y	FL, USA	Dry Tortugas National Park	18-May-22	Loggerhead Key	24.635007	-82.921341	*Thalassia testudinum*	
**Panacea**	**FL, USA**	**Florida Panhandle**	**10-May-23**	**Panacea**	**29.99911**	**-84.35056**	***Thalassia testudinum***	**OR879055**
MAIRIM	Puerto Rico	Jobos Bay National Estuarine Research Reserve	1-Feb-23	MAIRIM Site	Not available	Not available	*Halophila stipulacea*	
CPL	Puerto Rico	Jobos Bay National Estuarine Research Reserve	1-Feb-23	Cayo Puerca Lulu	Not available	Not available	*Halophila stipulacea*	
Z1	VA, USA	York River	2-Aug-17	York River State Park	Not available	Not available	*Zostera marina*	
Z2	MA, USA	West Falmouth Harbor	Not available	West Falmouth Harbor	Not available	Not available	*Zostera marina*	
Z3	AK, USA	Sitka	Not available	Sitka	Not available	Not available	*Zostera marina*	
Z4	New Zealand	Not available	Dec-22	New Zealand	Not available	Not available	*Zostera marina*	
Z5	Kalmar, Sweden	Not available	Not available	Kalmar	Not available	Not available	*Zostera marina*	
